# Clinical and Epidemiologic Characteristics of Hospitalized Patients with Laboratory-Confirmed Respiratory Syncytial Virus Infection in Eastern China between 2009 and 2013: A Retrospective Study

**DOI:** 10.1371/journal.pone.0165437

**Published:** 2016-11-01

**Authors:** Dawei Cui, Luzhao Feng, Yu Chen, Shengjie Lai, Zike Zhang, Fei Yu, Shufa Zheng, Zhongjie Li, Hongjie Yu

**Affiliations:** 1 Department of Laboratory Medicine, First Affiliated Hospital, College of Medicine, Zhejiang University, 79 Qingchun Road, Hangzhou, 310003, China; 2 Key Laboratory of Clinical In Vitro Diagnostic Techniques of Zhejiang Province, Hangzhou, 310003, China; 3 Division of Infectious Disease, Key Laboratory of Surveillance and Early-warning on Infectious Disease, Chinese Centre for Disease Control and Prevention, 155 Changbai Road, Beijing, 102206, China; 4 School of Public Health, Fudan University, Key Laboratory of Public Health Safety, Ministry of Education, 130 Dongan Road, Xuhui District, Shanghai, 200032, China; Kliniken der Stadt Köln gGmbH, GERMANY

## Abstract

Respiratory syncytial virus (RSV) is a leading cause of morbidity and mortality worldwide in children aged <5 years and older adults with acute lower respiratory infections (ALRIs). However, few studies regarding the epidemiology of hospitalizations for RSV infection have been performed previously in China. Here, we aimed to describe the clinical and epidemiologic characteristics of hospitalized patients with laboratory-confirmed RSV infection in eastern China. Active surveillance for hospitalized ALRI patients using a broad case definition based on symptoms was performed from 2009–2013 in 12 sentinel hospitals in eastern China. Clinical and epidemiologic data pertaining to hospitalized patients of all ages with laboratory-confirmed RSV infection by PCR assay were collected and analyzed in this study. From 2009 to 2013, 1046 hospitalized patients with laboratory-confirmed RSV infection were enrolled in this study, and 14.7% of patients had subtype A, 24.2% of patients had subtype B, 23.8% of patients with subtype not performed, and 37.3% of patients had RSV coinfections with other viruses. RSV and influenza coinfections (33.3%) were the most common coinfections noted in this study. Moreover, young children aged <5 years (89.1%, 932/1046), particularly young infants aged <1 year (43.3%, 453/1046), represented the highest proportion of patients with RSV infections. In contrast, older adults aged ≥60 years (1.1%, 12/1046) represented the lowest proportion of patients with RSV infections among enrolled patients. The peak RSV infection period occurred mainly during autumn and winter, and 57% and 66% of patients exhibited symptoms such as fever (body temperature ≥38°C) and cough separately. Additionally, only a small number of patients were treated with broad-spectrum antiviral drugs, and most of patients were treated with antimicrobial drugs that were not appropriate for RSV infection. RSV is a leading viral pathogen and a common cause of viral infection in young children aged <5 years with ALRIs in eastern China. Effective vaccines and antiviral agents targeting RSV are needed to mitigate its large public health impact.

## Introduction

Respiratory syncytial virus (RSV) is a leading cause of viral infection in children and older adults worldwide, particularly young children aged <5 years [1]. Studies indicate that RSV infection is associated with high morbidity and mortality rates in children with acute lower respiratory infections (ALRIs) in both industrialized and developing countries [1–4]. Approximately 34 million RSV-related ALRIs occur in children under 5 years of age, resulting in at least 3.4 million hospitalizations and 66,000–199,000 RSV-related deaths, 99% of which occur in developing countries [1]. RSV infection is responsible for approximately 1/4 of hospitalizations in children aged <5 years in China [2, 5–9] in areas such as Jingzhou [6], Lanzhou [7], Hangzhou [8], Suzhou [9], and Hong Kong [2].

Accumulating evidence indicates that RSV is a common cause of severe ALRIs, such as bronchiolitis and pneumonia, particularly in young children (aged <5 years) and older adults [10–12]. Safe and effective vaccines are needed to combat the high morbidity and mortality rates associated with RSV infection, and some candidates are in development. Previous reports have estimated the prevalence of RSV infection in hospitalized children and adults and determined that RSV seasons vary by geographic region and that rates of hospitalizations vary by age [13–16]. However, more data regarding the clinical and epidemiologic characteristics of RSV infection are needed to develop strategies to prevent and control the disease.

In this study, we aimed to evaluate the epidemiology, clinical characteristics and management of ALRIs caused by RSV infection among hospitalized patients of all age groups in eastern China between 2009 and 2013 in a hospital-based surveillance study.

## Methods and Materials

### Ethics Statement

RSV data collection from patients with ALRIs was considered public health surveillance by the National Health and Family Planning Commission of the People’s Republic of China. Brief verbal informed consent was obtained from all individuals enrolled in this study, according to the Declaration of Helsinki (1964), and this study was approved by the Ethics Committee of the Chinese Center for Disease Control and Prevention (China CDC, Beijing, China).

### Case Ascertainment

All subjects with laboratory confirmed RSV infection were hospitalized at 12 sentinel hospitals located in 6 provinces in eastern China between January 2009 and December 2013. Detailed data pertaining to these patients, including data regarding patient demographic and clinical characteristics and laboratory results, were obtained from an online data management system established by the China CDC [17–18]. Patient enrollment, sample collection, laboratory testing, and case reporting were conducted by all sentinel hospitals and laboratories according to a national surveillance protocol [17].

Patients were admitted to surveillance wards presided over by the departments of pediatrics, internal medicine, and infectious diseases or to the intensive care units (ICUs) of the abovementioned sentinel hospitals and screened by physicians and nurses for ALRI, which was diagnosed according to the following criteria: (1) at least one of the following manifestations of acute infection: fever (≥38°C), abnormal white blood cell (WBC) differentials, leukocytosis (WBC count more than 10,000/mL) or leukopenia (WBC count less than 4,000/mL), and chills; and (2) at least one of the following signs/symptoms of respiratory tract infection: cough, sputum production, shortness of breath, lung examination abnormalities (crackles or wheeze), tachypnea, and chest pain [17–18].

### Sample Collection and Testing

Clinical samples, such as nasopharyngeal swabs, aspirates and sputum, were collected and immediately placed in viral transport medium (VTM) before being stored at 4–8°Cat each sentinel hospital. These specimens were subsequently transferred to the nearest influenza network laboratory (provincial or prefecture Center for Disease Control and Prevention [CDC]) for diagnostic testing. Most of the specimens were tested within 24 hours of collection, and any specimens not tested within 24 hours were stored in VTM at -70°C. All specimens were tested for RSV (A and B subtypes), human influenza virus, human parainfluenza virus (HPIV), human bocavirus (HBoV), adenovirus (AdV), rhinovirus (RV), coronavirus (CoV), human metapneumovirus (HMPV), and enterovirus (EV)by real-time polymerase chain reaction (PCR), reverse transcriptase PCR (RT-PCR) or real-time RT-PCR, as previously described [17,19,20].

### Data Collection and Statistical Analysis

Detailed data regarding patient demographic and clinical characteristics and laboratory results were collected by the staff of the sentinel hospitals and laboratories using standardized case reporting forms and were uploaded into the online data management system. Descriptive statistics were used to analyze the demographic, epidemiological and clinical characteristics of the patients with RSV infection. Statistical analysis was performed with SPSS software (v18.0, SPSS, Chicago, IL, USA). Wilcoxon tests were used to compare the medians of continuous variables, and chi-square tests were performed to analyze frequency data. *P*<0.05 was considered statistically significant.

## Results

### Characteristics of RSV Patients

**Fig 1:**
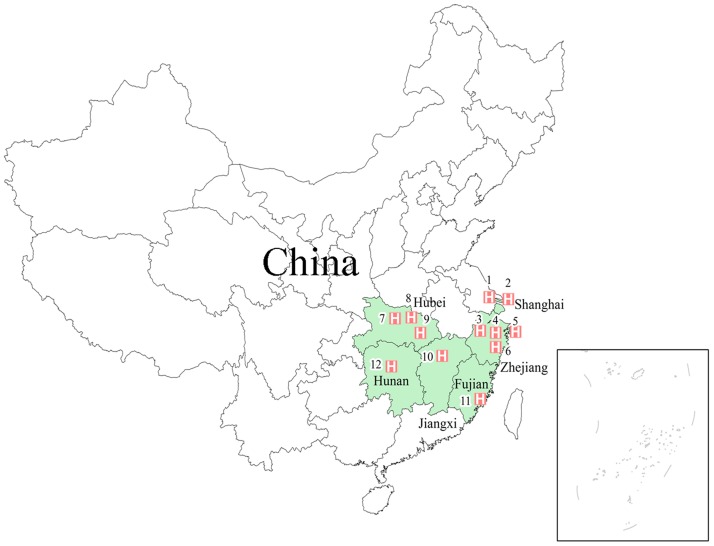
Locations of the 12 sentinel hospitals in eastern China at which respiratory syncytial virus (RSV) patients were treated. The red numbers indicate the locations of the surveillance sites. A total of 12 sentinel hospitals located in six provinces were enrolled in the final RSV infection analysis. 1). Children's Hospital of Shanghai, Shanghai Jiao Tong University; 2). Children’s Hospital of Fudan University; 3). Huzhou Central Hospital, Zhejiang Province; 4). The First Affiliated Hospital, School of Medicine, Zhejiang University; 5). The Children’s Hospital, School of Medicine, Zhejiang University; 6). Zhejiang Provincial Center for Disease Control and Prevention; 7). Renmin Hospital of Wuhan University; 8). Tongji Hospital, Tongji Medical College, Huazhong University of Science & Technology; 9). Wuhan Women and Children Medical Care Center, Hubei Province; 10). Jiangxi Provincial Children’s Hospital; 11). Maternal and Children's Health Hospital of Fujian Province; 12). Hunan Provincial People’s Hospital.

**Fig 2:**
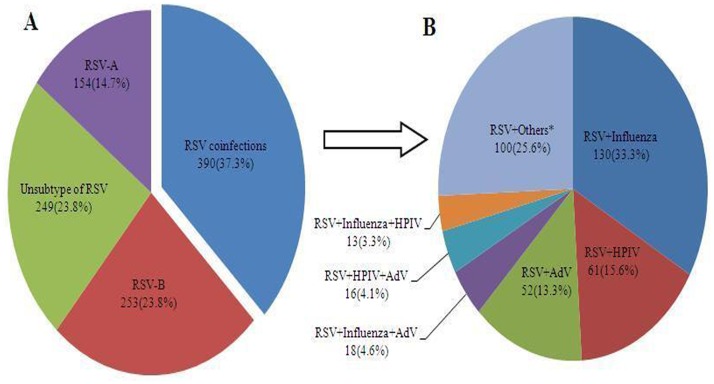
Patients with laboratory-confirmed RSV infection. (A) Distributions of RSV subtypes and coinfections among 1046 patients. (B) Distributions of other viral infections among 390 patients with RSV coinfections. *Others indicated RSV+HBoV+RV, RSV+EV, RSV+CoV, or RSV+HMPV, as less than 13 cases of each were noted. HPIV, human parainfluenza virus; HBoV, human bocavirus; AdV, adenovirus; RV, rhinovirus; CoV, coronavirus; HMPV, human metapneumovirus; EV, enterovirus.

From January 2009 to December 2013, 1046 hospitalized patients with laboratory-confirmed RSV infections were treated at 12 sentinel hospitals in 6 provinces in eastern China (Fig 1). Of these patients, 154 (14.7%) were diagnosed with RSV-A, 253 (24.2%) were diagnosed with RSV-B, 249 (23.8%) were subtyping not performed, and 390 (37.3%) were diagnosed with coinfections with RSV and other respiratory tract viruses (Fig 2A). Influenza virus (48.7%, 190/390) was the most common infectious agent noted in patients mixed infections, and coinfections with RSV/influenza (33.3%, 130/390) were the most frequent coinfections noted in this study, as 13 patients were diagnosed with RSV-A and influenza, 9 patients were diagnosed with RSV-B and influenza, and 108 patients were diagnosed with RSV subtyping not performed and influenza. Furthermore, 61 (15.6%) and 52 patients (13.3%) were diagnosed with RSV and HPIV or RSV and AdV coinfections, respectively (Fig 2B).

### Gender and Age Distributions of RSV Patients

In this study, 671 (64.1%) of the enrolled patients were male, and no significant differences were observed between men and women with the respect to the incidences of RSV-A and RSV-B infections and coinfections (Table 1). RSV infection was more common in young children aged <5 years (89.1%, 932/1046) than in patients aged ≥5 years (11.9%, 114/1046). RSV-A (85.1%, 131/154), RSV-B (90.9%, 230/253), subtyping not performed (87.1%, 217/249) and RSV coinfection (83.3%, 325/390) were also more common in young children than in other patients. Moreover, RSV infection was more common in young infants aged <6 months (26.8%, 280/1046) than in other patients. RSV-A (40.0%, 60/154), RSV-B (39.5%, 100/253), and subtyping not performed (22.1%, 55/249) were also more common in this age group than in other age groups. Additionally, the median age of patients with RSV single-infections (8–12 months) was considerably lower than that of patients with coinfections (24 months) (*P* = 0.000, Mann-Whitney Test). RSV infection exhibited a gradually decreasing trends with increasing age, as did RSV-A, RSV-B, and subtyping not performed and coinfections.

**Table 1:** Gender and age distributions of patients with laboratory-confirmed RSV infection.

Characteristics	Total (n = 1046)	RSV-A (n = 154)	RSV-B (n = 253)	Subtyping not performed (n = 249)	Coinfections (n = 390)
Male	671 (64.1%)	96 (62.3%)	156 (61.7%)	168 (67.5%)	251 (64.3%)
Female	375 (35.9%)	58 (37.7%)	97 (38.3%)	81 (32.5%)	139 (35.6%)
Age (Years, M, Q_R_)	1 (0.4–3)	0.8 (0.2–1)	0.8 (0.3–2)	1 (0.5–3)	2 (0.8–3)
Age group					
0–5 m	280 (26.8%)	60 (40.0%)	100 (39.5%)	55 (22.1%)	65 (16.7%)
6–11 m	173 (16.5%)	29 (18.8%)	42 (16.6%)	53 (21.3%)	49 (12.5%)
1 y	199 (19.0%)	27 (17.5%)	45 (17.8%)	51 (20.5%)	76 (19.5%)
2 y	98 (9.4%)	6 (3.9%)	13 (5.1%)	25 (10.0%)	54 (13.8%)
3 y	97 (9.3%)	4 (2.6%)	17 (6.7%)	23 (9.2%)	53 (13.6%)
4 y	56 (5.4%)	5 (3.2%)	13 (5.1%)	10 (4.0%)	28 (7.2%)
5 y	29 (2.8%)	1 (0.6%)	4 (1.6%)	7 (2.8%)	17 (4.4%)
6–9 y	66 (6.3%)	3 (1.9%)	13 (5.1%)	12 (4.8%)	36 (9.2%)
10–14 y	16 (1.5%)	2 (1.3%)	1 (0.4%)	7 (2.8%)	7 (1.8%)
15–59 y	20 (1.9%)	10 (6.5%)	1 (0.4%)	5 (2.0%)	5 (1.3%)
≥60 y	12 (1.1%)	7 (4.5%)	4 (1.6%)	1 (0.4%)	0 (0%)

### Temporal/Seasonal Trends of RSV

**Fig 3:**
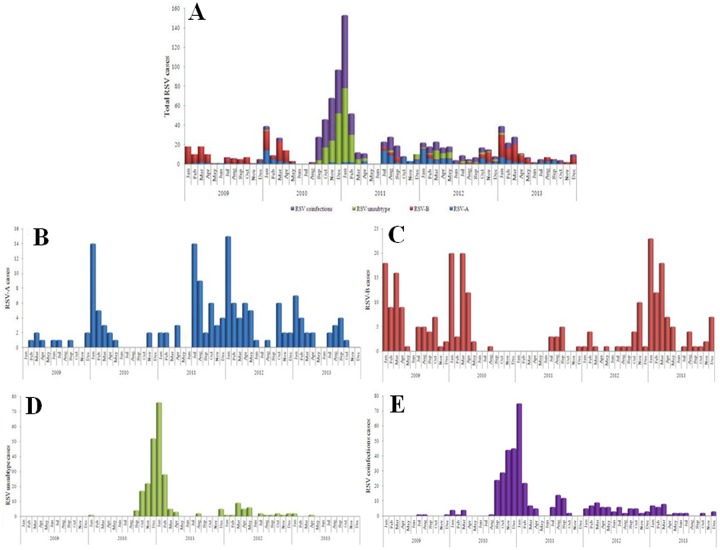
Seasonal trends of laboratory-confirmed RSV infection in hospitalized patients in eastern China between 2009 and 2013. (A) RSV infection; (B) RSV-A; (C) RSV-B; (D) Subtyping not performed; (E) Coinfection with other viruses.

RSV infection exhibited clear seasonal trends during this study. The majority of RSV infections occurred between September 2010 and February 2011 (Fig 3A). RSV-A infections were most frequently observed in spring, autumn and winter from January to March 2010, July 2011 to May 2012, and January to February 2013 (Fig 3B). Similarly, RSV-B infections were most frequently observed between January and April in 2009, 2010 and 2013 (Fig 3C). Moreover, RSV with subtyping not performed and RSV coinfections were most frequently observed between October 2010 and February 2011 (Fig 3D) and between September 2010and February 2011 (Fig 3E) compared with other seasons, respectively.

### Clinical Characteristics of RSV Patients

A temperature of ≥38°C was documented in 61.0% of patients with RSV infection, and 59.0% of patients with RSV coinfection with other viruses at the time of physical examination. A moderately significant difference in temperature was observed between patients with RSV coinfections and patients with RSV single-infections (χ2 = 4.825, *P* = 0.028). Cough was the most common symptom exhibited by RSV patients (66.3%), and runny nose (22.7%) and sputum production (18.6%) were also common among affected patients. Moreover, significant differences between patients with RSV coinfections with other viruses and patients with RSV single-infections were observed with respect to symptoms such as cough (χ2 = 5.774, *P* = 0.016), runny nose (χ^2^ = 8.749, *P* = 0.003), sore throat (χ^2^ = 10.357, *P* = 0.001), sputum production (χ2 = 8.063, P = 0.005), shortness of breath (χ2 = 27.700, P = 0.000), and difficulty breathing (χ2 = 5.920, P = 0.015), as well as WBC count (χ2 = 0.015, P = 0.903). Moreover, the patients with RSV and influenza coinfection had highest rate in clinical symptoms as sore throat (18.4%), shortness of breath (8.4%) and/or difficulty breathing (11.1%). Of 803 patients who underwent a chest X-ray, 168 (20.9%) were found to have radiographic evidence of pneumonia, although the proportion of the patients with pneumonia/RSV coinfection was not significantly different from that of patients with RSV single-infection (χ2 = 0.653, P = 0.419) (Table 2).

**Table 2:** Clinical characteristics of patients with laboratory-confirmed RSV infection.

Characteristics	Total (n = 1046)	RSV-A (n = 154)	RSV-B (n = 253)	Subtyping not performed (n = 249)	Coinfection (n = 390)	
					Influenza (n = 190)	No Influenza (n = 200)
**Symptoms and Signs**						
Fever ≥38°C	571/1003 (56.9%)	80 (51.9%)	119 (47.0%)	142 (57.0%)	113 (59.4%)	117 (55.4%)
Cough	694 (66.3%)	124 (80.5%)	215 (85.0%)	114 (45.8%)	103 (54.2%)	138 (69%)
Runny nose	237 (22.7%)	52(33.8%)	73 (28.9%)	43 (17.3%)	32 (16.8%)	37 (18.5%)
Sore throat	93 (8.9%)	11 (7.1%)	3 (1.2%)	30 (12.0%)	35 (18.4%)	14 (7%)
Sputum production	195 (18.6%)	39 (25.3%)	25 (9.9%)	41 (16.5%)	31 (16.3%)	59 (29.5%)
Shortness of breath	37 (3.5%)	2 (1.3%)	2 (0.8%)	4 (1.6%)	16 (8.4%)	13 (6.5%)
Difficulty breathing	55 (5.3%)	9 (5.8%)	7 (2.8%)	10 (4.0%)	21 (11.1%)	8 (4%)
Headache	71 (6.8%)	18 (11.7%)	4 (1.6%)	19 (7.6%)	12 (6.3%)	18 (9%)
Fatigue	33 (3.2%)	10 (6.5%)	7 (2.8%)	6 (2.4%)	8 (4.2%)	2 (1%)
Abdominal pain	5 (0.4%)	2 (1.3%)	0	0	0	3 (1.5%)
Diarrhea	13 (1.2%)	5 (3.2%)	4 (1.6%)	0	0	4 (2%)
**Clinical Examinations**						
Lung auscultation	122/603 (20.2%)	19 (12.3%)	46 (18.2%)	12 (4.8%)	12 (6.3%)	33 (16.5%)
Abnormal X-ray	168/803 (20.9%)	36 (23.4%)	65 (25.7%)	9 (3.6%)	21 (11.1%)	37 (18.5%)
**Laboratory Examinations**						
**Blood routine numbers**	**453**	**69**	**175**	**41**	**91**	**77**
WBC (×10^9^/L)	8.8 (6.4–12.14)	8.3 (6.6–12.12)	8.6 (6.4–10.5)	10.3 (6.6–14.4)	9.3 (6.75–13.25)	9.13 (6.39–12.34)
<4.0	17 (3.7%)	1 (1.4%)	12 (6.9%)	1 (2.4%)	1 (1.1%)	3 (3.9%)
>10.0	173 (38.2%)	27 (39.1%)	52 (29.7%)	22 (53.7%)	40 (44.0%)	32 (41.6%)
L(%)	43 (27–58)	48 (33–58)	50 (36–64)	32(19–52)	32 (20–53)	42 (26–54)
<20%	71 (15.7%)	5 (7.2%)	15 (8.6%)	12 (29.3%)	23 (25.3%)	15 (19.5%))
>40%	257 (56.7%)	45 (65.2%)	118 (67.4%)	16 (39.0%)	39 (42.9%)	39(50.6%)
N(%)	44 (28–60)	39 (27–54)	38 (25–53)	55 (36–74)	55 (34–71)	49 (35–67)
<40%	200 (44.2%)	47 (68.1%)	121 (69.1%)	17 (41.5%)	37 (40.7%)	40 (51.9%)
>70%	67 (14.8%)	5 (7.2%)	11 (6.3%)	11 (26.8%)	26 (28.6%)	14 (18.2%)
PLT (×10^9^/L)	313 (223–409)	336 (223–436)	338 (248–429)	252 (202–413)	272 (210–376)	295 (219–378)
<100	7 (1.5%)	1 (1.4%)	1 (0.6%)	1 (2.4%)	1 (1.1%)	3 (3.9%)
>300	237 (52.3%)	38 (55.1%)	105 (60.0%)	18 (43.9%)	41 (45.1%)	35 (45.5%)
Hb (g/L)	119 (109–129)	116 (104–128)	116 (108–130)	120 (112–128)	118 (113–126)	120 (111–128)
<110	137 (30.2%)	26 (37.7%)	51 (29.1%)	8 (19.5%)	28 (30.8%)	24 (31.2%)
>150	13 (2.9%)	5 (7.2%)	2 (1.1%)	3 (7.3%)	2 (2.2%)	1 (1.3%)
**Blood gas analysis (cases)**	**104**	**24**	**21**	**8**	**10**	**41**
pH	7.38 (7.34–7.41)	7.36 (7.33–7.4)	7.37 (7.34–7.4)	7.41 (7.39–7.45)	7.37 (7.33–7.39)	7.38 (7.34–7.42)
<7.35	35 (33.6%)	9 (37.5%)	8 (38.1%)	1 (12.5%)	3 (30%)	14 (34.1%)
>7.45	13 (12.5%)	2 (8.3%)	1 (4.8%)	2 (25.0%)	0	8 (19.5%)
PCO_2_(mmHg)	31 (25–36)	31 (26–38)	34 (28–38)	32 (28–41)	29 (20–33)	30 (24–33)
<35	77 (74.0%)	15 (62.5%)	15 (71.4%)	4 (50.0%)	8 (80%)	35 (85.4%)
>45	8 (7.7%)	2 (8.3%)	2 (9.5%)	1 (12.5%)	1 (10%)	2 (4.9%)
PO_2_(mmHg)	83 (67–100)	78 (65–97)	91 (64–99)	86 (65–99)	83 (76–102)	82 (67–99)
<80	45 (43.3%)	14 (58.3%)	10 (47.6%)	4 (50.0%)	3 (30%)	14 (34.1%)
>100	26 (25.0%)	5 (20.8%)	5 (23.8%)	2 (25.0%)	3 (30%)	11 (26.8%)
SO_2_(%)	96 (92–98)	95 (91–97)	97 (92–98)	96 (93–98)	96 (94–97)	96 (93–98)
<93	29 (27.9%)	10 (41.7%)	8 (38.1%)	1 (12.5%)	0	10 (24.4%)
>98	14 (13.5%)	2 (8.3%)	4 (19.0%)	2 (25.0%)	1 (10%)	5 (12.2%)

### Analysis of Drug Administration

Currently, only a few drugs exist effective against RSV infections or other viral infections. Many antimicrobial drugs were used by doctors to treat patients in this study. Of 147 patients with RSV infection, 102 were treated with one drug. Amoxicillin-clavulanate (23.5%, 24/102) was the most frequently prescribed antibacterial drug, and Cefuroxime (17.6, 18/102) was the second most frequently prescribed antibacterial drug. Seventy patients were treated with two drugs with Azithromycin (16.7%, 12/72) and Cefuroxime (13.9%, 10/72) that were the most frequently prescribed drugs. Moreover, multiple other drugs were also prescribed for patients. However, broad-spectrum antiviral agents, such as Ribavirin and Potassium sodium dehydroandroandrographolide succinate, were not commonly prescribed to patients with RSV infection. Additionally, some drugs were used excessively and administered for longer time periods than recommended, such as Azithromycin (whose course is normally limited to 5 days) and Cefuroxime (whose course is normally limited to 7 days) (Table 3).

**Table 3:** Drugs administered to 147 patients with laboratory-confirmed RSV infection.

Drugs	Number of drug administrations			
	One (n = 102)	Two (n = 72)	Three (n = 24)	≥Four (n = 4)
**Antimicrobial drugs**				
Amoxicillin-clavulanate acid	24	7	1	0
Azithromycin	9	12	2	1
Cefotaxime	10	4	4	0
Meropenem	1	0	0	1
Penicillin	1	0	1	0
Cefuroxime	18	10	3	0
Cefmetazole	2	1	0	0
Cefminox	3	0	0	0
Cefoperazonesulbactam	11	5	2	1
Ceftriaxone	5	8	1	1
Ceftazidime	5	0	0	0
Cefotiam	1	1	1	0
Ceftizoxime	7	1	0	0
Imipenem/cilastatin	1	1	0	0
Ampicillin sulbactam	2	0	1	0
Acetyl midecamycin	0	3	3	0
Mezlocillin	0	1	2	0
Moxifloxacin	0	1	0	0
Cetirizine	0	1	0	0
Teicoplanin	0	3	1	0
Latamoxef	0	2	0	0
Piperacillin/tazobactam	0	1	0	0
Ampicillin	0	1	0	0
Salbutamol	0	0	1	0
Erythromycin	0	0	1	0
**Drugs for supportive therapy**	0	0	0	0
Terbutaline sulfate	1	4	0	0
**Broad-spectrum antiviral drugs**	0	0	0	0
Potassium sodium dehydroandroandrographolide succinate	1	4	0	0
Ribavirin	0	1	0	0

## Discussion

This study is a part of a hospital-based ALRI surveillance study in China and documented the etiologies, epidemiology and clinical characteristics of ALRIs caused RSV, which was diagnosed via laboratory testing, using data from a sentinel surveillance system in eastern China. From 2009 to 2013, a total of 1046 hospitalized ALRI patients (children and adults) were treated at 12 sentinel hospitals in eastern China. We found that most patients had RSV single-infections and that 37.3% had mixed viral infections caused by RSV and other respiratory tract viruses. Additionally, we observed that coinfections with RSV and influenza were the most frequently diagnosed mixed infections among our patient population, a finding consistent with those of previous studies in China [17–21] and other countries [15, 22–26]. Taken together, these findings indicate that coinfections may influence severe illnesses in patients with RSV infections, especially in young children aged <5 years.

Although many studies regarding RSV have focused on children aged <5 years and elderly adults, among whom the morbidity and mortality rates of RSV infection are high, little is known regarding the effects of RSV among patients of all ages. We analyzed the overall age distributions of the patients with RSV infections. Our results indicated that children aged <5 years represented the largest proportion of patients with RSV infections (86.4%) and coinfections (83.3%), particularly young children (total: 62.3%; coinfections: 48.7%) aged <2 years, compared to patients aged ≥5 years. These observations indicate that an association exists between RSV prevalence and age, a finding consistent with those of previous reports in China, Japan, Thailand and other countries [17–21, 24–27].

Our surveillance data indicate that RSV infection occurred almost year-round from 2009 to 2013 and peaked mainly during autumn and winter in areas of eastern China with subtropical climates, results similar to those reported previously in China and other countries [19–24] but different from those reported in tropical countries [16]. These findings indicate that RSV infection is characterized by a seasonality that may be associated with regional climate and demographic factors [6,12,22]. The seasonality of RSV is different in tropical and subtropical countries; thus, hospitals in these countries can utilize seasonally timed interventions and specific vaccination and hospital infection control strategies [1,9,13].

Most of the patients enrolled in this study had symptoms of fever (temperature ≥38°C) and cough, and moderately significant differences in temperature and cough were observed between patients with RSV coinfections and patients with RSV single-infections. These observations are consistent with those of previous reports [17–19]. A few patients experienced shortness of breath or difficulty breathing, the majority of whom suffered from RSV coinfections, indicating that RSV coinfections with other viruses can cause more severe disease than RSV single-infections. However, the proportions of patients with pneumonia were not significantly different between patients with RSV coinfection and patients with RSV single-infection, indicating that pneumonia development was likely related to other factors, such as patient age, patient physical condition, and viral load [23–28]. Generally, viral infections do not significantly affect common laboratory parameters (such as WBC, PO_2_, and PCO_2_), particularly during their early stages [11, 29–30]. Our data indicated that there were no significant differences in laboratory parameters between patients with RSV single-infections and patients with coinfections.

Currently, only a few antivirals exist that are effect against RSV infections and other viral infections, and delays in viral testing have important effects on infection severity, particularly among patients with RSV, influenza virus and enterovirus infections [1,11,30]. In our study, only a few patients with RSV infections were treated with broad-spectrum antiviral drugs (Ribavirin and Potassium sodium dehydroandroandrographolide succinate) that were not specific for RSV. Most of the antimicrobial drugs administered to patients in this study were not appropriate for patients with RSV infection but are commonly used in such patients in China. It is well known that antimicrobial drug overuse selects for highly resistant organisms, causes immune dysfunction, and increases viral loads, which may explain the high morbidity and mortality rates associated with RSV infection [12, 26–28].

Our study had some limitations. First, patients were tested for RSV and other respiratory tract viruses, but not bacterial pathogens; thus, our data regarding pathogens causing ALRIs were not comprehensive. Second, we tested for RSV-A and RSV-B to determine the relationships between RSV subtype and age, seasonality and clinical characteristics, but some of total RSV infections did not test for RSV subtypes. Third, some of our clinical and laboratory data were not comprehensive, which affected our ability to estimate RSV disease severity.

## Conclusion

In summary, this study provided important epidemiologic information regarding RSV and associated respiratory viral infections in patients with ALRIs in eastern China. RSV infection is common in young children, and implementing disease control strategies (such as safe vaccination) during the appropriate seasons may have a significant impact on public health. Clinical and laboratory data pertaining to patients with RSV infection may be helpful for optimizing patient treatments. Timely and accurate diagnosis of RSV infection in patients with ALRIs is necessary to prevent large-scale RSV spread and to reduce the burden imposed by the disease and the misuse of antimicrobial drugs. These preliminary results indicate that more surveillance data are needed to estimate the RSV disease burden and to determine whether geographic locations, climate, other environmental factors and RSV coinfections impact the severity ofRSV-related epidemics.
